# Cyclic AMP-CRP Modulates the Cell Morphology of *Klebsiella pneumoniae* in High-Glucose Environment

**DOI:** 10.3389/fmicb.2019.02984

**Published:** 2020-01-21

**Authors:** Long Liu, Feiyu Li, Li Xu, Jingjie Wang, Moran Li, Jie Yuan, Hui Wang, Ruiping Yang, Bei Li

**Affiliations:** ^1^School of Basic Medical Sciences, Hubei University of Medicine, Shiyan, China; ^2^Hubei Key Laboratory of Embryonic Stem Cell Research, Hubei University of Medicine, Shiyan, China; ^3^Biomedical Research Institute, Hubei University of Medicine, Shiyan, China

**Keywords:** *Klebsiella pneumoniae*, cyclic AMP-CRP, cell morphology, glucose, regulation

## Abstract

Bacteria can modify their morphology in response to environmental stimuli for survival or host defense evasion. The rich glucose *in vivo* or in the Luria–Bertani (LB) medium shortened the cell length of *Klebsiella pneumoniae*. The environmental glucose decreased the levels of cyclic AMP (cAMP) and the transcription of *crp*, which declined the cAMP–cAMP receptor protein (cAMP-CRP) activity. The cell length of *crp* deletion mutant was significantly shorter than that of the wild type (0.981 ± 0.057 μm vs. 2.415 ± 0.075 μm, *P* < 0.001). These results indicated that the high environmental glucose alters the bacterial morphology to a round form through regulating the activity of cAMP-CRP complex. Comparative proteomics analysis showed increased expression of 10 proteins involved in cell division or cell wall biosynthesis in the *crp* deletion strain. Five of them (*omp*A, *tol*B, *ybg*C, *fts*I, and *rcs*F) were selected to verify their expression in the high-glucose environment, and overexpression of *tol*B or *rcs*F shortened the bacterial length similar to that of the *crp* deletion strain. Electrophoretic mobility shift assay indicated that CRP directly negatively regulates the transcription of *tol*B and *rcs*F by binding to the promoter regions. This study first proved the role and partial regulation mechanism of CRP in altering cell morphology during infection and provided a theoretical basis for elucidating the mechanism in diabetes mellitus susceptible to *K. pneumoniae*.

## Introduction

*Klebsiella pneumoniae* is a rod-shaped, Gram-negative bacterium that belongs to the family Enterobacteriaceae and is widely distributed in the mouth, skin, intestines, hospital settings, and medical devices. This bacterium is an opportunistic pathogen responsible for many nosocomial infections ranging from urinary tract infection to pneumonia. Over the past years, cases of primary liver abscesses (PLAs) and other invasive infections, such as meningitis, necrotizing fasciitis, and endophthalmitis, which are caused mainly by the hypermucoviscous phenotype of *K. pneumoniae*, have increased worldwide (Fang et al., 2007; Siu et al., 2012; Zhang et al., 2016; Lee et al., 2017a). Patients with diabetes have high susceptibility to *K. pneumoniae* infections (Yang et al., 2009; Lin et al., 2013c; Lee et al., 2017b). *K. pneumoniae* strains are more virulent in diabetic mice than in normal ones (Wu and Tsai, 2005). Exogenous glucose could stimulate the production of CPS and type 3 fimbriae, the virulence factors of *K. pneumoniae* (Lin et al., 2013b, 2016). These processes are regulated by the global regulator cyclic AMP (cAMP) receptor protein (CRP) and cAMP-CRP signaling pathway. The supply of environmental glucose can inhibit the production of the intracellular second messenger cAMP and inactivate the cAMP-CRP signaling pathway (Lin et al., 2013b, 2016).

Bacterial morphology distinguishes bacterial species and regulates bacterial attachment and pathogenicity (Huang et al., 2008; Yang et al., 2016). For *Helicobacter pylori*, mutants with non-helical cells exhibit stomach colonization defects in a murine colonization model (Bonis et al., 2010; Sycuro et al., 2010). Bacteria can modify their morphology in response to their host environmental stimuli or different growth conditions (Justice et al., 2004). However, no reports were found about the alteration mechanism of *K. pneumoniae* morphology. Nutritional status is a factor favoring bacterial shape modification that affects the nutrient acquisition (van Teeseling et al., 2017). The effect of high glucose in diabetic patients on the cell morphology of *K. pneumoniae* NTUH-2044, a capsular serotype K1 strain with hypermucoviscosity phenotype, is still unknown.

In this study, we determined the effects of environmental glucose on the cell morphology of *K. pneumoniae* and the partial underlying regulatory mechanism of cAMP-CRP for the cell morphology. We revealed that the abundant glucose in type 2 diabetes mellitus (T2DM) mice model or in the LB medium altered the length of *K. pneumoniae* cells. The in-frame deletion of the *crp* gene caused similar changes in the bacterial morphology. Comparative proteomic analysis between the wild-type (WT) and *crp* knockout strains showed the upregulated expression of 10 genes associated with cell wall synthesis and division in the *crp* knockout strain. Five of them were selected out to verify their expression in the high-glucose environment and the expressions of *tol*B, *fts*I, and *rcs*F increased. Overexpression of TolB or RcsF but not FtsI affected the bacterial morphology. In addition, the CRP binding sites on the promoter regions of *tol*B and *rcs*F genes were found and cAMP-CRP complex could directly regulate the proteins expression. These results indicated that the cAMP-CRP pathway is a key regulatory pathway that regulates cell shape to correspond to the environmental glucose changes, and *tol*B and *rcs*F play roles in this processing through direct regulation by cAMP-CRP complex. This study provides further understanding on the pathogenesis of *K. pneumoniae* in patients with diabetes and one of the regulation mechanisms of bacterial morphological alteration.

## Materials and Methods

### Bacterial Strains and Growth

The bacterial strain *K. pneumoniae* NTUH-2044, a capsular serotype K1 strain with hypermucoviscosity phenotype, was isolated from a liver abscess patient in Taiwan (Chou et al., 2004). For general *K. pneumoniae* cultivation, the bacteria were cultured with shaking in LB broth at 37°C without or with 12 mM glucose, the critical blood glucose concentration of patients with diabetes (Quincozes-Santos et al., 2017).

### Construction of the Mouse Model of T2DM and Infection With *K. pneumoniae*

Four-week-old male c57bl/6 mice were randomly divided into two groups and fed with basal diet or high-fat diet (40% of total energy from fat) for 1 month, then the T2DM mice were intraperitoneally injected with Streptozocin (Sigma, Japan), which dissolved by 0.1 M sodium citrate for five continuous days (30 mg/kg body weight, one injection/day) to induce the pancreatic cell damage (Zhang et al., 2008). Control group mice were injected with 0.1 M sodium citrate buffer. After 2 weeks, the mice were kept fasting for 14 h, and then, the fasting blood glucose levels were measured by withdrawing blood samples. After intraperitoneal injection with 10^5^ colony forming units (CFU) *K. pneumoniae* for 20 h, the liver tissue was taken out for homogenization and stained with crystal violet.

### Construction of *crp* Deletion Mutant and Complementation of *K. pneumoniae*

An in-frame deletion strain Δ*crp* and a complementary strain C*-crp* were previously constructed using an allelic-exchange strategy (Ou et al., 2017).

### Bacterial Staining and Scanning Electron Microscopy of *K. pneumoniae* Cells and Quantitative Morphology Analyses

Bacterial strains were cultured in late logarithmic period. Then, 10 μl of fluids was coated onto slides and stained with crystal violet. For the scanning electron microscopy of *K. pneumoniae*, the bacteria were coated with palladium gold using a Hummer 6.2 sputter coater (Anatech USA, Hayward, CA, United States) and observed by a VEGA 3 LMU high-resolution scanning electron microscope (TESCAN, Czech) at 30 kV. Images of the bacteria were quantitatively analyzed by ImageJ (version 1.52a) with NeuronJ plugin and Skeleton tool. Bacterial length was estimated using the central axis length calculated for 16–50 cells/strain.

### Proteomic Sample and Peptide Preparation

The WT and Δ*crp* strains were cultured in LB medium overnight, followed by 100-fold dilution with fresh LB broth. The bacteria were harvested when OD600 reached ∼1.2, and the bacterial pellets were washed twice with phosphate-buffered saline. The bacterial proteins were then extracted using a bacterial protein extraction kit (BestBio, Shanghai, China) according to the manufacturer’s instructions. Protein concentrations were determined using the bicinchoninic acid assay method (Smith et al., 1985). Protein samples were digested using the filter-aided sample preparation method (Wisniewski et al., 2009). Each protein extraction (200 μg) was mixed with 4 μl of tris(2-carboxyethyl)phosphine reducing reagent and incubated at 60°C for 1 h. Then, 2 μl of methyl methanethiosulfonate cysteine-blocking reagent was added and incubated at room temperature for 10 min. After the protein solutions were transferred to a 10-K ultrafiltration tube and centrifuged at 12,000 × *g* for 20 min at 4°C, urea (8 M, pH 8.5) was added and centrifuged under the same conditions. This step was repeated twice. Then, tetraethylammonium bromide (TEAB) (0.25 M, pH 8.5) was added and centrifuged three times for 20 min. After the tube was replaced with a new collection tube, 50 μl of TEAB trypsin (0.5 M, trypsin/protein = 1:50) were added, and the mixture was incubated at 37°C overnight. Then, trypsin (trypsin/protein = 1:100) was added and incubated at 37°C for 4 h, followed by centrifugation at 12,000 × *g* for 20 min. Finally, 50 μl of 0.5 M TEAB was mixed with the collected sample and centrifuged at 12,000 × *g* for 4 min at 4°C. The enzymatically peptides were collected at the bottom of the tube.

### LC-MS/MS Protein Identification

The iTRAQ reagents 114, 115, 118, and 121 were used to label the peptide samples from the WT or Δ*crp* strain. The labeled peptides were separated using the LC-20AB high-performance liquid chromatography pump system (Shimadzu, Kyoto, Japan). Analytical separations were performed using the LC-20AD nano-high-performance liquid chromatography (Shimadzu, Kyoto, Japan) coupled with a Triple TOF 5600 System (AB SCIEX, Concord, ON, Canada). The liquid chromatography with tandem mass spectrometry (LC-MS/MS) data were matched using the Mascot search engine against the *K. pneumoniae* database to identify and quantify the proteins. A coefficient of variation was calculated to remove the poorly reproducible proteins, and coefficient of variation ≤0.5 indicated high repeatability. *t-*Test was used to identify significant (*P* < 0.05) differences in the means between the cultivated Δ*crp* and WT. The results were considered statistically significant if a twofold change was observed in the protein expression levels.

### Bioinformatics Analysis

Gene Ontology (GO) annotation^1^ and Kyoto Encyclopedia of Genes and Genomes pathway^2^ enrichment analysis were used to determine the functional subcategories and metabolic pathways for the differentially expressed proteins. The mass spectrometry results were analyzed by the R project, and the enriched GO categories were analyzed by the REVIGO tool^3^ (Supek et al., 2011). STRING 9.1 was used to explore the interaction network and functional relations in the differential expression of proteins. IPA (Version 01-04, QIAGEN, United States) was used to analyze the regulation effects, and Cytoscape (version 3.8) was used to generate the interaction map.

### RNA Isolation and Quantitative Real-Time PCR

*Klebsiella pneumoniae* NTUH-K2044 cultured overnight were 100-fold diluted in fresh LB broth containing 0 mM glucose, 12 mM glucose, or 12 mM glucose +500 nM cAMP to grow to late logarithmic period. Total RNA was isolated using a RNeasy Mini column (Qiagen) according to the manufacturer’s recommendations. Chromosomal DNA was eliminated from the purified RNA by RNase-free DNase I (Qiagen) treatment, and complementary DNA was synthesized. The RNA was reverse transcribed with a SuperScript^TM^ III first-stand synthesis system (Invitrogen) using arbitrary oligonucleotide primers in 20 μl of the reaction mixture. Quantitative real-time PCR (qRT-PCR) was performed in a Light Cycler CFX96 instrument (Bio-Rad). A Light Cycler Taq Man Master kit was used to detect the expression of genes. Relative genes expression was quantified using the comparative threshold cycle 2^–ΔΔCT^ method with 16S rRNA as the endogenous reference.

### Construction of the Overexpressed Gene Strains

The *tol*B, *fts*I, and *rcs*F genes were, respectively, amplified by PCR using the corresponding primers listed in Supplementary Table S1 and inserted into the km-pGEM-T-easy plasmid (Bryan et al., 2011). The recombinant plasmids were transformed into the WT strain by electrotransformation. The transcription of genes in the different strains was quantified by qRT-PCR using the strain with empty km-pGEM-T-easy as the control.

### lacZ Fusion and β-Galactosidase Activity Assay

The promoter-proximal DNA region of *tol*B, *fts*I, or *rcs*F was cloned into the low-copy-number transcriptional fusion vector pHRP309 harboring a promoter-less lacZ reporter gene (Parales and Harwood, 1993). The *K. pneumoniae* WT and Δ*crp* strains transformed with the recombinant plasmids were grown to measure the β-galactosidase activity in the cellular extracts using a β-galactosidase enzyme assay system (Miller, 1972).

### Electrophoretic Mobility Shift Assay

The *crp* gene was inserted into the downstream of a sequence encoding hexa-histidine in pET28a so that *Escherichia coli* host produces His_6_-CRP, and then, the recombinant protein was purified using a Ni–NTA agarose column (Spriestersbach et al., 2015). The putative promoter region fragments of *tol*B, *fts*I, or *rcs*F were amplified and labeled using the biotin 3′ end DNA labeling kit (Beyotime, China). The labeled single-stranded probes were annealed with an annealing reagent (Beyotime) and incubated with increasing amounts of His_6_-CRP protein (0, 0.6, 1, and 2 μM). After incubation at room temperature for 30 min, the mixtures were analyzed using 6% native polyacrylamide gel containing 1 nmol cAMP. The biotin-labeled DNA was detected by the Chemiluminescence EMSA Kit (Thermo Fisher Scientific, United States).

### Cyclic AMP Concentration Assay

*Klebsiella pneumoniae* were cultured LB medium with or without 12 mM glucose to late logarithmic period and were adjusted to 10^7^ CFU/ml. Then, the bacteria were washed twice with phosphate-buffered saline and resuspended in 1 × lysis buffer (CST, United States) and lysed by sonication for 10 min. The lysate was briefly centrifuged at 14,000 rpm for 10 min. After collecting the supernatant, cAMP concentration was detected by the cyclic AMP XP^®^ Chemiluminescent Assay Kit (CST, #8019S) according to the manufacturer’s introductions (Lin et al., 2013a).

### Statistical Analysis

Experiments were performed with three independent replicates, and data were statistically analyzed by GraphPad Prism (version 5.0, United States). The values were expressed as mean ± standard deviation. Significant differences of the bacterium lengths between the groups were calculated by unpaired two-tailed Student’s *t*-test, quantitative PCR, and β-galactosidase activity data were analyzed by one-way ANOVA, and *P* < 0.05, *P* < 0.01, and *P* < 0.001 all indicate statistical significance.

## Results

### High-Glucose Environment Affects the Morphology of *K. pneumoniae*

**FIGURE 1:**
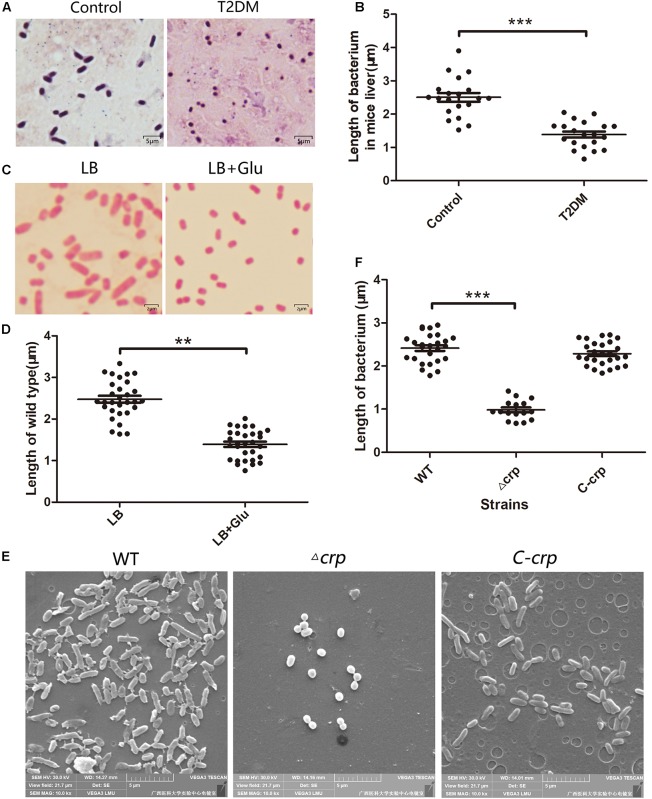
Glucose addition or *crp* knockout affected the morphology of *Klebsiella pneumoniae* NTUH-K2044. **(A)** The morphology of the WT strain in the infected mice liver without or with type 2 diabetes was observed after Gram-staining. Mice infects with *K. pneumoniae* by intraperitoneal injection. T2DM, type 2 diabetes mellitus mice, control indicates healthy mice. **(B)** The lengths of *K. pneumoniae* in **(A)** were analyzed by ImageJ plugin. **(C)** The morphology of the WT strain cultured in LB medium without or with 12 mM glucose was observed using a microscope by 1,000 × magnification. **(D)** The lengths of strains were analyzed by ImageJ. **(E)** Scanning electron micrographs of WT, Δ*crp*, and C*-crp* strains. **(F)** Quantitative analysis of the lengths of different strains of *K. pneumoniae.* WT, wild type; Δ*crp*, *crp* deletion strain; C*-crp*, *crp* complementary strain. Error bars represent standard deviation. ^∗∗^*P* < 0.01; ^∗∗∗^*P* < 0.001; for comparison between each group with wild type as calculated by unpaired two-tailed Student’s *t-*test.

Diabetic patients are highly susceptible to *K. pneumoniae* infection and resulting in PLAs (Fang et al., 2007; Zhang et al., 2016). In this study, T2DM mice model was constructed to evaluate the physiological and pathological changes after infection with WT *K. pneumoniae*. All the T2DM mice died within 2 days after intraperitoneal injection with 10^5^ CFU bacteria, whereas the control group survived (data not shown). The liver tissues from T2DM and control group were taken out and observed after Gram staining. Most of bacteria in the control group were rod-like shape while shortened to a spherical or short rod-like shape in T2DM mice (Figure 1A), and the average length of *K. pneumoniae* in control and T2DM mice liver was 2.506 ± 0.131 μm and 1.387 ± 0.089 μm, respectively (Figure 1B). To explore the effects of glucose on the growth and length of *K. pneumoniae*, the WT bacteria were cultured in LB medium with or without 12 mM glucose. The majority of WT bacteria had substantially changed morphology in the glucose-rich LB medium relative to that in the control LB medium (Figure 1C). The WT *K. pneumoniae* cultured in LB was rod-like shape and became spherical after adding 12 mM glucose (Figure 1C). The average length of the WT strain in LB medium supplemented with 12 mM glucose was shorter than that in LB medium without glucose (1.654 ± 0.064 μm vs. 2.475 ± 0.083 μm, *P* ≤ 0.05) (Figure 1D).

### Carbon Metabolism Regulator CRP Could Regulate Bacterial Morphology

CRP is a global regulatory protein activated by cAMP, the second messenger repressed by the environment glucose. We further observed the change in the morphology of Δ*crp* strain. Δ*crp* and C*-crp* strains were previously constructed as described (Ou et al., 2017). The morphologies of Δ*crp*, C*-crp*, and WT strains were scanned by an electron microscope. As shown in Figure 1E, the majority of the WT and C*-crp* strains presented a rod-like shape, whereas the Δ*crp* strain appeared shorter or globular. The length of the Δ*crp* strain was evidently shorter than that of the WT (0.981 ± 0.057 μm vs. 2.415 ± 0.066 μm, *P* ≤ 0.05), the difference between C*-crp* and WT was not statistically significant (Figure 1F). These results indicated that CRP played a role in maintaining the cell morphology of *K. pneumoniae.*

### Comparative Proteomic Analysis

**FIGURE 2:**
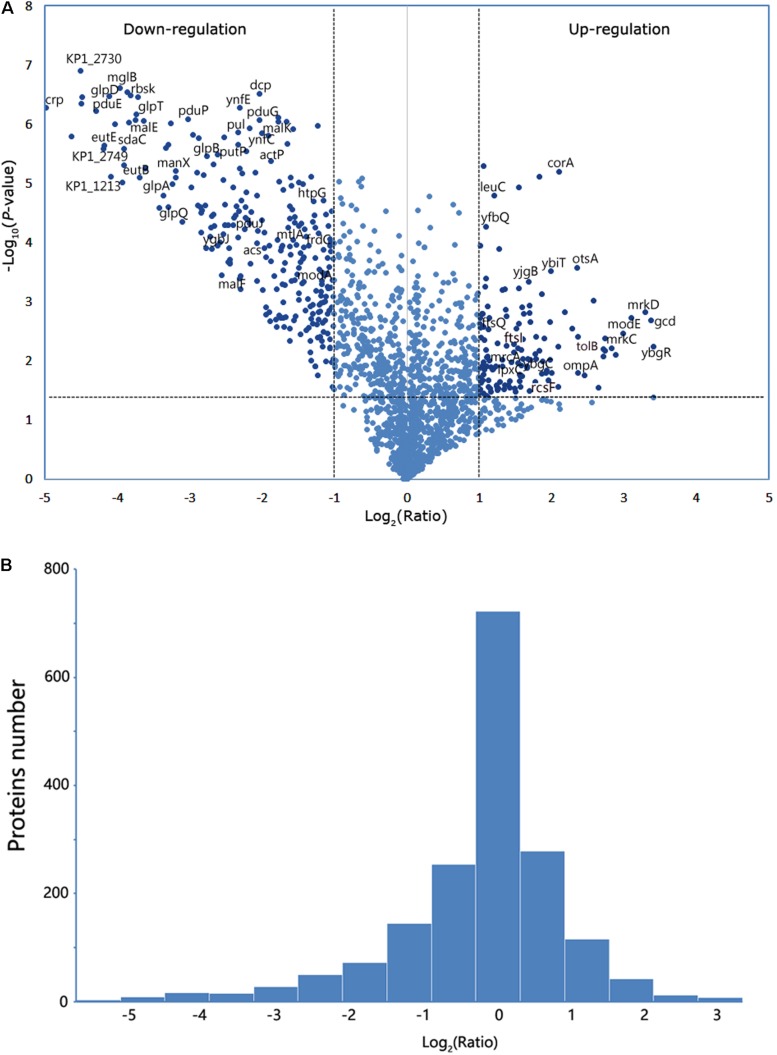
Analysis of the mass spectrometry data. **(A)** Volcano plot from the mass spectrometry data demonstrates the magnitude and significance of the cellular proteins from the deletion strain compared with the control (WT). Horizontal dashed line shows where *p*-value is 0.05 [-log_10_ (0.05) = 1.3], and the vertical dashed lines show where the fold change is 2 [log_2_ (2) = 1] or 0.5 [log_2_ (0.5) = -1]. The twofold change and *p*-value of 0.05 were used as the threshold cutoff. **(B)** The number of proteins was counted according to the fold change of log_2_ (ratio).

The proteins from the WT and its isogenic mutant Δ*crp* were extracted and quantitatively examined by LC-MS/MS analyses to globally define the target proteins that are regulated by CRP and eventually affect the bacterial morphology. Changes in the two sources of protein profiles were analyzed, and total 1,032 proteins were identified by Swiss-Prot database. Among these proteins, 426 exhibited a twofold-change difference (*P* ≤ 0.05) from two biological replicates according to the identification parameters. The data were further organized in a volcano plot (Figure 2A). The expression level of 146 proteins increased by more than twofold, and the level of 280 proteins decreased to <0.5-fold in the *crp* knockout bacteria compared with that in the WT strain (Figure 2B). Therefore, these proteins with altered expression levels were possibly regulated directly or indirectly by the CRP. The quantification data and deferentially expressed proteins are listed in the Supplementary Table S2.

### Bioinformatics Analysis on the Cell Division and Cell Wall Biosynthesis of Related Proteins

**FIGURE 3:**
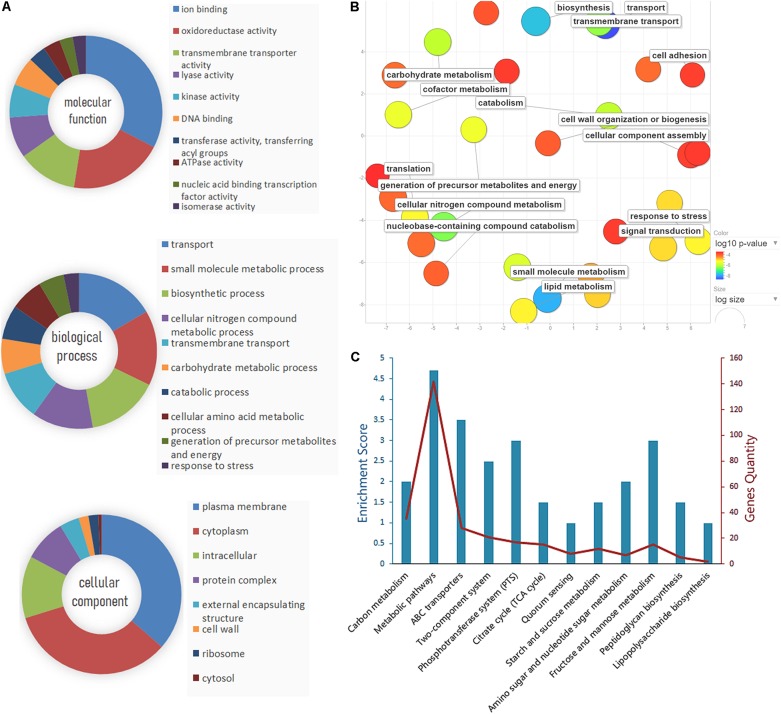
Gene Ontology (GO) analysis and pathway enrichment analysis of the differential expression proteins. **(A)** GO annotation of the differential proteins identified by liquid chromatography with tandem mass spectrometry (LC-MS/MS), including molecular function, biological process, and cellular component. **(B)** The enriched GO categories were analyzed by the REVIGO tool. The colored spots showed the regulatory proteins which appeared to be commonly involved in the major biological functions. **(C)** Pathway enrichment and gene quantity analysis of differentially expressed proteins.

The identified proteins were analyzed using GO term analysis to obtain the global view of the biological processes and molecular functions regulated by CRP. The major biological processes, cellular components, and molecular functions are shown in Figure 3A. According to the biological processes, the identified proteins were classified into several major classes. The enriched GO categories were then submitted to the REVIGO website to reduce the untrustworthy proteins (Supek et al., 2011). The result (Figure 3B) showed that part of proteins are associated with cell wall biosynthesis, cellular component assembly, and cell adhesion. In addition, the differential expressed proteins contributed to biological processes were further investigated by Kyoto Encyclopedia of Genes and Genomes pathway enrichment analysis (Figure 3C). The metabolic pathways, ABC transporters, phosphotransferase system, and two-component system were the major pathways. Some proteins are also involved in the pathways associated with lipopolysaccharide and peptidoglycan biosynthesis.

**FIGURE 4:**
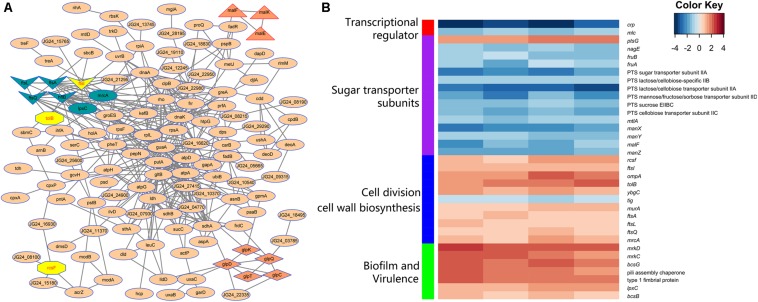
Analysis of the indicated proteins in the cell division, transport, and metabolism. **(A)** Analysis of the protein–protein interaction of the identified proteins by STRING 9.0 and generated by Cytoscape 3.8. Medium confidence was set and disconnected nodes were hided. Nodes are proteins, and lines represent functional associations between proteins. Gene clusters associated with cell division (dark blue and yellow), maltose (triangle), and glycerol (diamond) metabolism involved in the network. **(B)** Functional clustering analysis of the differential proteins identified in the *crp* deletion and WT strain of *K. pneumoniae*.

STRING database was used to detect the functional relations, generate the interaction networks (Figure 4A) and further understand the functional interactions of the twofold differential proteins. The network analyses showed a few differential genes involved in cell division, maltose and glycerol uptake, and metabolism. The proteomic data showed a cluster of proteins that were tightly associated with the biosynthesis of cell wall component. Further clustering analysis indicated that the proteins involved in sugar transport, cell division, or cell wall biosynthesis, and the virulence factor were differentially expressed after *crp* been deleted (Figure 4B). The levels of most proteins associated with cell division and cell wall biosynthesis, including RcsF, FtsI, FtsA, FtsL, FtsQ, OmpA, TolB, YbgC, MurA, and MrcA, were upregulated, and these proteins may be regulated by CRP. The functions and fold changes of the identified genes involved in cell division and cell wall biosynthesis are listed in Table 1. RcsF functions in the signal transduction from the cell surface to Rcs signaling system (Castanie-Cornet et al., 2006; Sato et al., 2017), while OmpA, which acts as an outer membrane (OM) porin, can form channels through the cellular membranes (Hong et al., 2006). TolB plays a role in OM invagination during cell constriction in the *trans-*envelope Tol-pal system (Zhuang et al., 2002; Gerding et al., 2007; Ridley and Lakey, 2015). The Fts opera, containing FtsA, FtsI, FtsL, and FtsQ, are involved in cell division (Chen and Beckwith, 2001; Kureisaite-Ciziene et al., 2018; Du et al., 2019). MrcA and MurA play a role in cell wall formation (Kock et al., 2004; Zhu et al., 2012), Tig is involved in protein export, and the only downregulated protein in the 11 proteins related to cell division (Martinez-Hackert and Hendrickson, 2009).

**TABLE 1:** Functions of genes involved in cell division and cell wall biosynthesis.

Locus_tag	Gene name	Gene Function	Fold change^a^
KP1_1055	*rcs*F	Outer membrane lipoprotein	3.415925562
KP1_1958	*omp*A	Outer membrane protein 3a	5.465537071
KP1_1702	*tol*B	Translocation protein TolB precursor	6.554541469
KP1_1697	*ybg*C	Tol-pal system-associated acyl-CoA thioesterase	3.258493781
KP1_3464	*fts*I	Division-specific transpeptidase, penicillin-binding protein 3 (PBP3)	2.931135476
KP1_0916	*fts*A	ATP-binding cell division protein	2.040040225
KP1_0905	*fts*L	Cell division protein	2.08796376
KP1_0915	*fts*Q	Membrane anchored protein involved in growth of wall at septum	2.179539979
KP1_5097	*mrc*A	Peptidoglycan bifunctional penicillin-binding protein 1a (PBP1a)	2.989608765
KP1_4910	*mur*A	UDP-*N*-acetylglucosamine 1-carboxyvinyltransferase	2.439517468
KP1_1269	*tig*	Trigger factor	0.47762087

*^*a*^Fold change indicates the expression ratio of wild type with *crp* mutant identified by quantitative mass spectrometry.*

### qRT-PCR Analysis Revealed That High-Glucose Environment Affects the Expression Levels of Related Genes

**FIGURE 5:**
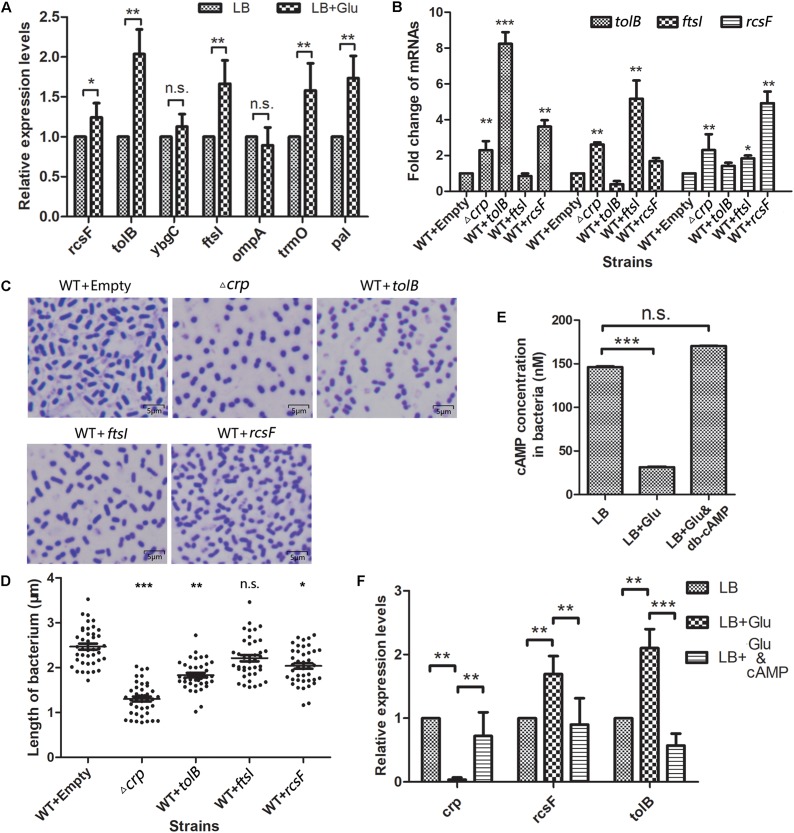
Selected genes in the WT *K. pneumoniae* affected the cell morphology. **(A)** The messenger RNA (mRNA) levels of five selected genes and *tol*A, *pal*, and *trm*O were detected by quantitative PCR after the wild-type (WT) strain was cultured in the glucose-rich medium. Comparison was performed by one-way ANOVA. **(B)** Quantitation of the indicated mRNA levels in WT strains overexpressed with the selected proteins. WT transformed with empty vector acted as the negative control. Comparison was performed by one-way ANOVA. **(C)** The morphology of the overexpressed strains and WT of *K. pneumoniae* was scanned by a microscope after staining with crystal violet. **(D)** The lengths of the WT, Δ*crp*, and overexpression strains were analyzed using ImageJ. Comparison between each group with WT as calculated by unpaired two-tailed Student’s *t-*test. **(E)** The *in vivo* cAMP concentrations of WT strain cultured in LB broth with or without 12 mM glucose or 12 mM glucose +500 nM cAMP analog dibutyryl-cAMP (db-cAMP) were measured. Error bars represent standard deviation. Comparison was performed by one-way ANOVA. **(F)** The mRNA levels of *crp*, *rcs*F, and *tol*B in WT strain cultured in LB with 0 mM glucose, 12 mM glucose, and 12 mM glucose combined with 500 nM db-cAMP were quantified. Comparison was performed by one-way ANOVA. Error bars represent standard deviation. ^∗^*P* < 0.05; ^∗∗^*P* < 0.01; ^∗∗∗^*P* < 0.001; n.s., not significant.

Here, the most significant upregulated genes (*omp*A, *tol*B, *ybg*C, and *rcs*F), which increased to more than threefold, and *fts*I, as the highest fold in the Fts opera, were selected as the targets to verify whether the environmental glucose changed the bacterial morphology due to the expression changes of these genes. The messenger RNA (mRNA) levels of these five genes were quantitatively detected in *K. pneumoniae* cultured without or with 12 mM glucose. The qPCR results (Figure 5A) showed that the expression levels of *rcs*F, *tol*B, and *fts*I but not *omp*A and *ybg*C increased substantially in the LB medium with 12 mM glucose than that in medium without glucose. Meanwhile, *pal* and *trm*O located in the downstream of *tol*B and *rcs*F also increased their expression in the glucose-rich conditions compared with in LB broth (Figure 5A).

### Overexpression of *rcs*F and *tol*B Affects Bacterial Morphology

Then, the relationship between the TolB, FtsI, or RcsF and the change in the bacterial morphology was determined. The genes encoding the indicated proteins were cloned into the km-pGEM-T-easy plasmid. The recombinant plasmids were transformed into the WT *K. pneumoniae* to construct the corresponding protein overexpression strains. The mRNA levels of *rcs*F, *tol*B, and *fts*I in the different strains were quantified by real-time PCR. The results showed that the mRNA levels of these genes were higher in the Δ*crp* mutant than in the WT strain, similar to the results of proteome analysis (Figure 5B). CRP negatively controlled the expression of these genes. The strains with the overexpression plasmids increased the expression level of the corresponding gene significantly. The shapes of the strains were observed by microscopy (Figure 5C), and the results showed that TolB (1.832 ± 0.049 μm) or RcsF (2.037 ± 0.062 μm) overexpression considerably shortened the bacterium length compared with the empty km-pGEM-T-easy plasmid group. Overexpressed FtsI also partially changed the bacterial shape, but the result was not statistically significant (2.213 ± 0.070 μm vs. 2.469 ± 0.066 μm) (Figure 5D).

### Glucose Affects the Intracellular cAMP Level and the *crp*, *tol*B, and *rcs*F Transcription

*In vitro*, external glucose decreased the intracellular cAMP level and *crp* transcription but increased the transcription levels of *rcs*F and *tol*B in *K. pneumoniae* (Figures 5E,F), and adding 500 nM exogenous cAMP analogs (db-cAMP) in the present of glucose increased the cAMP level equivalent to the glucose-free condition (Figure 5E). Meanwhile, cAMP addition increased the transcription of *crp* and declined the *rcs*F and *tol*B transcription (Figure 5F).

### CRP Directly Regulates the Transcription of *rcs*F and *tol*B

**FIGURE 6:**
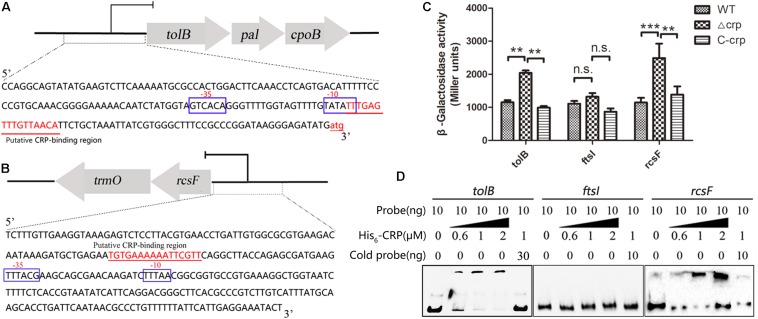
cAMP-CRP binding directly to *tol*B and *rcs*F promoter was identified by EMSA and *lac*Z fusion assay. **(A)** The *tol*B*-pal-cpo*B operon and the promoter sequence. The large arrows represent the open-reading frames, and the CRP binding site was marked as red color and underlined. **(B)** The *rcs*F*-trm*O operon and the promoter sequence. **(C)** *Lac*Z fusion assay of the cAMP-CRP binding to the *tol*B and *fts*I promoter regions. Recombinant pHRP309 plasmids were transferred into the WT, *crp*-knockout, and *crp* complementary mutant of *K. pneumoniae*. Error bars represent standard deviation. ^∗∗^*P* < 0.01; ^∗∗∗^*P* < 0.001; n.s., not significant; comparison was performed by one-way ANOVA. **(D)** cAMP-CRP binding directly to *tol*B and *rcs*F promoter regions was identified by EMSA. Ten nanogram unlabeled probe (cold probe) was added to compete with labeled probe for binding His_6_-CRP protein.

The mechanisms of CRP in the regulation of *tol*B and *rcs*F that led to the change in bacterial morphology were studied by performing *lac*Z fusion β-galactosidase activity assay and EMSA. The putative CRP binding site located at the promoter region of indicated opera were observed in the upstream of *tol*B and *rcs*F genes via bioinformatics analysis (Figures 6A,B), but the *fts*I gene was not found. Then, the gene fragments containing each promoter were cloned into the promoter-less *Lac*Z fusion vector pHRP309 to generate the *tol*B*–lac*Z, *fts*I*–lac*Z, or *rcs*F*–lac*Z fusion plasmids. The recombinant plasmids were transferred into the WT, Δ*crp* mutant, and C-*crp* strains. The results showed that Δ*crp* carrying the *tol*B*–lac*Z or *rcs*F*–lac*Z fusion vector caused higher activity of β-galactosidase compared with the WT and C-*crp* group, while *fts*I*–lac*Z did not remarkably change the level of β-galactosidase (Figure 6C). Then, the recombinant His_6_-CRP protein was expressed and purified from *E. coli* BL21(DE3) to verify the results of *Lac*Z reporter assay, and EMSA was performed using the labeled *tol*B, *fts*I, or *rcs*F promoter probes. The results showed that binding of His_6_-CRP could be observed after incubation with *tol*B and *rcs*F promoter, while *fts*I promoter did not form a complex with His_6_-CRP (Figure 6D). These results indicated that CRP could bind directly to the predicted CRP binding sites and regulated their transcription.

## Discussion

Diabetes is a well-known risk factor for the development of *Klebsiella* liver abscess (Fang et al., 2007; Lee et al., 2016). The impaired host defense mechanisms in DM may be highly associated with PLA (Muller et al., 2005). Patients with controlled glycemia and immune function exhibit an improved state and tend to have lower PLA rate than those with uncontrolled glycemia (Lin et al., 2013c). In addition to the influence of host immune system in DM, several studies have proved that exogenous glucose can stimulate the production of virulence factors of *K. pneumoniae* (Lin et al., 2013b, 2016). This phenomenon suggests that a high-glucose environment enhances the pathogenicity of bacteria, which may also contribute to the high susceptibility to *K. pneumoniae* infection in DM.

Pathogens can change their cell morphology to survive in diverse environments and evade host defenses (Li and Nielsen, 2017; Rodriguez et al., 2019). In *Enterobacteriaceae*, the cell size diversity helps bacteria to avoid complement-mediated killing and play a role in their pathogenesis (Huang et al., 2008; Dalia and Weiser, 2011). In this study, the length of *K. pneumoniae* was shortened in T2DM mice and the LB medium with 12 mM glucose, the critical blood glucose concentration of patients with diabetes. One factor that contributed to the shortened cell length of *K. pneumoniae* may be the increased bacterial growth rate in the glucose-rich medium, which affects the cell division and cell wall synthesis. The *crp* knockout strain, lack of the catabolite repressor protein, had shorter cell lengths with decreased growth rate, suggesting that CRP played an important regulation role in the diversity of *K. pneumoniae* morphology, and the alter of cell shape in rich glucose environment was not just due to the increasing of growth rate. The rich environmental glucose inactivated the cAMP-CRP regulatory complex. Compared with the Δ*crp* strain which completely lacks the regulation role of cAMP-CRP, WT in the glucose-rich medium still forms the cAMP-CRP complex, which regulates the downstream genes. This may explain the shorter cell lengths in the Δ*crp* strain than those of WT in the glucose-rich medium.

In most bacteria, the cell size and shape are determined by the cell wall. The cell wall of Gram-negative bacteria is composed of two membranes, namely, the inner membrane and OM. The space between inner membrane and OM defines the periplasm, a cellular compartment that contains a thin layer of peptidoglycan. Many proteins associated with the peptidoglycan synthesis or cell division, and the proteins or lipoproteins on the OM had been proved to influence the bacterial morphology (Cabeen and Jacobs-Wagner, 2005; Jorgenson et al., 2014; Heller et al., 2017). Comparative proteomics analysis revealed that CRP regulates 11 proteins, which involved in cell division or cell wall biosynthesis. However, several proteins, such as FtsZ and MreB, which related to the maintenance of cell shape, had not been identified due to the limitation of peptide preparation and protein identification. Five proteins were selected for detailed analysis, and the cell lengths of strains overexpressing RcsF or TolB were shortened similar to the bacteria cultured in the glucose-rich medium. RcsF is an OM lipoprotein component of the Rcs system, a stress-signaling cascade complex involving at least six components (Wall et al., 2018). RscF is the sensor which detects the damage caused by chemicals targeting to the OM or peptidoglycan and activates the RcsB regulator of the system (Guo and Sun, 2017). The phosphorylated RcsB can positively regulate the FtsZ gene involved in the cell division (Konovalova et al., 2016; Guo and Sun, 2017). The FtsZ-mediated Z-ring force determines the direction of the cell wall growth and indirectly determines the cell shape (Cabeen and Jacobs-Wagner, 2005). TolB is a soluble periplasmic protein in the Tol-Pal complex, the conserved complex of cell envelopes in Gram-negative bacteria (Lazzaroni et al., 2002). The mutant genotypes of the Tol-Pal proteins vary in length and exhibit some chain reaction compared with the WT in *Salmonella typhimurium* (Masilamani et al., 2018). In *K. pneumoniae*, the lengths of each *tol*B or *rcs*F overexpression strains were longer than those of the Δ*crp* strain, indicating that the alteration of cell length in Δ*crp* was through regulating multiple genes, not just one gene, to adapt to the environment. The high-level glucose decreases the cAMP-CRP activity, thus reversing the repression of *rcs*F and *tol*B genes. The *rcs*F and *tol*B expression levels change altered the cellular morphology by direct or indirect regulation of other genes associated with cell shape.

In summary, this study used comparative proteomic analysis to examine how glucose-regulating genes affect the bacterial morphology. After comparing the proteomes of Δ*crp* and WT strains, *tol*B, *fts*I, and *rcs*F were selected to verify their effects on the bacterial shape regulated by cAMP-CRP complex. Overexpression of TolB or RcsF shortened the bacterial length similar to that of *crp*-deleted strain. CRP negatively regulates the transcription of *tol*B and *rcs*F by directly binding to their promoter regions. Thus, the results implied that high environmental glucose repressed the cAMP-CRP level to regulate the cell morphology of *K. pneumoniae* and the expression of *tol*B and *rcs*F, the genes play roles in the alteration of bacterial morphology via direct regulation by cAMP-CRP complex. This study first proved the role and partial regulation mechanism of CRP altering cell morphology in *K. pneumoniae*. The clinical importance of cell shape alteration during *K. pneumoniae* infection will be explored in future studies.

## Data Availability Statement

The datasets generated for this study can be found in the iProX (ID: PXD015322).

## Ethics Statement

The animal study was reviewed and approved by the Laboratory Animal Ethics Committee of Hubei University of Medicine.

## Author Contributions

LL and BL designed the research project and wrote the manuscript. LL and FL performed all major experiments. LL and LX analyzed the MS data and purified the protein. JW and ML finished the microscopy scanning. JY and HW constructed the recombinant plasmids. RY performed the statistical analyses. BL provided general supervision of the project.

## Conflict of Interest

The authors declare that the research was conducted in the absence of any commercial or financial relationships that could be construed as a potential conflict of interest.

## Footnotes

1 http://www.geneontology.org/

2 http://www.genome.jp/kegg/

3 http://revigo.irb.hr/revigo.jsp

## References

[B1] Bonis M. Ecobichon C. Guadagnini S. Prevost M. C. Boneca I. G. (2010). A M23B family metallopeptidase of *Helicobacter pylori* required for cell shape, pole formation and virulence. *Mol. Microbiol.* 78 809–819. 10.1111/j.1365-2958.2010.07383.x 20815828

[B2] Bryan S. J. Burroughs N. J. Evered C. Sacharz J. Nenninger A. Mullineaux C. W. (2011). Loss of the SPHF homologue Slr1768 leads to a catastrophic failure in the maintenance of thylakoid membranes in Synechocystis sp. PCC 6803. *PLoS One* 6:e19625. 10.1371/journal.pone.0019625 21625427 PMC3100299

[B3] Cabeen M. T. Jacobs-Wagner C. (2005). Bacterial cell shape. *Nat. Rev. Microbiol.* 3 601–610. 10.1038/nrmicro1205 16012516

[B4] Castanie-Cornet M. P. Cam K. Jacq A. (2006). RcsF is an outer membrane lipoprotein involved in the RcsCDB phosphorelay signaling pathway in *Escherichia coli*. *J. Bacteriol.* 188 4264–4270. 10.1128/JB.00004-06 16740933 PMC1482940

[B5] Chen J. C. Beckwith J. (2001). FtsQ, FtsL and FtsI require FtsK, but not FtsN, for co-localization with FtsZ during *Escherichia coli* cell division. *Mol. Microbiol.* 42 395–413. 10.1046/j.1365-2958.2001.02640.x 11703663

[B6] Chou H. C. Lee C. Z. Ma L. C. Fang C. T. Chang S. C. Wang J. T. (2004). Isolation of a chromosomal region of *Klebsiella pneumoniae* associated with allantoin metabolism and liver infection. *Infect. Immun.* 72 3783–3792. 10.1128/iai.72.7.3783-3792.2004 15213119 PMC427404

[B7] Dalia A. B. Weiser J. N. (2011). Minimization of bacterial size allows for complement evasion and is overcome by the agglutinating effect of antibody. *Cell Host Microbe* 10 486–496. 10.1016/j.chom.2011.09.009 22100164 PMC3222866

[B8] Du S. Henke W. Pichoff S. Lutkenhaus J. (2019). How FtsEX localizes to the Z ring and interacts with FtsA to regulate cell division. *Mol. Microbiol.* 112 881–895. 10.1111/mmi.14324 31175681 PMC6831102

[B9] Fang C. T. Lai S. Y. Yi W. C. Hsueh P. R. Liu K. L. Chang S. C. (2007). *Klebsiella pneumoniae* genotype K1: an emerging pathogen that causes septic ocular or central nervous system complications from pyogenic liver abscess. *Clin. Infect. Dis.* 45 284–293. 10.1086/519262 17599305

[B10] Gerding M. A. Ogata Y. Pecora N. D. Niki H. de Boer P. A. J. (2007). The trans-envelope Tol-Pal complex is part of the cell division machinery and required for proper outer-membrane invagination during cell constriction in E-coli. *Mol. Microbiol.* 63 1008–1025. 10.1111/j.1365-2958.2006.05571.x 17233825 PMC4428343

[B11] Guo X. P. Sun Y. C. (2017). New Insights into the non-orthodox two component rcs phosphorelay system. *Front. Microbiol.* 8:2014. 10.3389/fmicb.2017.02014 29089936 PMC5651002

[B12] Heller D. M. Tavag M. Hochschild A. (2017). CbtA toxin of *Escherichia coli* inhibits cell division and cell elongation via direct and independent interactions with FtsZ and MreB. *PLoS Genet* 13:e1007007. 10.1371/journal.pgen.1007007 28931012 PMC5624674

[B13] Hong H. Szabo G. Tamm L. K. (2006). Electrostatic couplings in OmpA ion-channel gating suggest a mechanism for pore opening. *Nat. Chem. Biol.* 2 627–635. 10.1038/nchembio827 17041590

[B14] Huang K. C. Mukhopadhyay R. Wen B. Gitai Z. Wingreen N. S. (2008). Cell shape and cell-wall organization in Gram-negative bacteria. *Proc Natl Acad Sci U.S.A.* 105 19282–19287. 10.1073/pnas.0805309105 19050072 PMC2592989

[B15] Jorgenson M. A. Chen Y. Yahashiri A. Popham D. L. Weiss D. S. (2014). The bacterial septal ring protein RlpA is a lytic transglycosylase that contributes to rod shape and daughter cell separation in *Pseudomonas aeruginosa*. *Mol. Microbiol.* 93 113–128. 10.1111/mmi.12643 24806796 PMC4086221

[B16] Justice S. S. Hung C. Theriot J. A. Fletcher D. A. Anderson G. G. Footer M. J. (2004). Differentiation and developmental pathways of uropathogenic *Escherichia coli* in urinary tract pathogenesis. *Proc Natl Acad Sci U.S.A.* 101 1333–1338. 10.1073/pnas.0308125100 14739341 PMC337053

[B17] Kock H. Gerth U. Hecker M. (2004). MurAA, catalysing the first committed step in peptidoglycan biosynthesis, is a target of Clp-dependent proteolysis in Bacillus subtilis. *Mol. Microbiol.* 51 1087–1102. 10.1046/j.1365-2958.2003.03875.x 14763982

[B18] Konovalova A. Mitchell A. M. Silhavy T. J. (2016). A lipoprotein/beta-barrel complex monitors lipopolysaccharide integrity transducing information across the outer membrane. *eLife* 5:e15276. 10.7554/eLife.15276 27282389 PMC4942254

[B19] Kureisaite-Ciziene D. Varadajan A. McLaughlin S. H. Glas M. Monton Silva A. Luirink R. (2018). Structural Analysis of the Interaction between the Bacterial Cell Division Proteins FtsQ and FtsB. *MBio* 9:e1346-18. 10.1128/mBio.01346-18 30206170 PMC6134095

[B20] Lazzaroni J.-C. Dubuisson J.-F. Vianney A. (2002). The Tol proteins of *Escherichia coli* and their involvement in the translocation of group A colicins. *Biochimie* 84 391–397. 10.1016/s0300-9084(02)01419-0 12423782

[B21] Lee C. R. Lee J. H. Park K. S. Jeon J. H. Kim Y. B. Cha C. J. (2017a). Antimicrobial resistance of hypervirulent *Klebsiella pneumoniae*: epidemiology, hypervirulence-associated determinants, and resistance mechanisms. *Front. Cell Infect. Microbiol.* 7:483. 10.3389/fcimb.2017.00483 29209595 PMC5702448

[B22] Lee I. R. Sng E. Lee K. O. Molton J. S. Chan M. Kalimuddin S. (2017b). Comparison of diabetic and non-diabetic human leukocytic responses to different capsule types of *Klebsiella pneumoniae* responsible for causing pyogenic liver abscess. *Front. Cell Infect. Microbiol.* 7:401. 10.3389/fcimb.2017.00401 28936426 PMC5594087

[B23] Lee I. R. Molton J. S. Wyres K. L. Gorrie C. Wong J. Hoh C. H. (2016). Differential host susceptibility and bacterial virulence factors driving Klebsiella liver abscess in an ethnically diverse population. *Sci. Rep.* 6:29316. 10.1038/srep29316 27406977 PMC4942785

[B24] Li Z. Nielsen K. (2017). Morphology changes in human fungal pathogens upon interaction with the host. *J. Fungi.* 3:66. 10.3390/jof3040066 29333431 PMC5753168

[B25] Lin C. T. Chen Y. C. Jinn T. R. Wu C. C. Hong Y. M. Wu W. H. (2013a). Role of the cAMP-dependent carbon catabolite repression in capsular polysaccharide biosynthesis in *Klebsiella pneumoniae*. *PLoS One* 8:e54430. 10.1371/journal.pone.0054430 23408939 PMC3569464

[B26] Lin T. H. Huang S. H. Wu C. C. Liu H. H. Jinn T. R. Chen Y. (2013b). Inhibition of *Klebsiella pneumoniae* Growth and Capsular Polysaccharide Biosynthesis by Fructus mume. *Evid. Based Complement Alternat. Med.* 2013:621701. 10.1155/2013/621701 24062785 PMC3770061

[B27] Lin Y. T. Wang F. D. Wu P.-F. Fung C. P. (2013c). *Klebsiella pneumoniae* liver abscess in diabetic patients: association of glycemic control with the clinical characteristics. *BMC Infect. Dis.* 13:56. 10.1186/1471-2334-13-56 23363608 PMC3568401

[B28] Lin C. T. Lin T. H. Wu C. C. Wan L. Huang C. F. Peng H. L. (2016). CRP-Cyclic AMP Regulates the Expression of Type 3 Fimbriae via Cyclic di-GMP in *Klebsiella pneumoniae*. *PLoS One* 11:e0162884. 10.1371/journal.pone.0162884 27631471 PMC5025149

[B29] Martinez-Hackert E. Hendrickson W. A. (2009). Promiscuous substrate recognition in folding and assembly activities of the trigger factor chaperone. *Cell* 138 923–934. 10.1016/j.cell.2009.07.044 19737520 PMC2799252

[B30] Masilamani R. Cian M. B. Dalebroux Z. D. (2018). *Salmonella* Tol-Pal Reduces Outer Membrane Glycerophospholipid Levels for Envelope Homeostasis and Survival during Bacteremia. *Infect. Immun.* 86:e173-18. 10.1128/IAI.00173-18 29735519 PMC6013679

[B31] Miller J. H. (1972). *Experiments in Molecular Genetics.* Cold Spring Harbor: Cold Spring Harbor Laboratory Press.

[B32] Muller L. M. Gorter K. J. Hak E. Goudzwaard W. L. Schellevis F. G. Hoepelman A. I. M. (2005). Increased risk of common infections in patients with type 1 and type 2 diabetes mellitus. *Clin. Infect. Dis.* 41 281–288. 10.1086/431587 16007521

[B33] Ou Q. Fan J. Duan D. Xu L. Wang J. Zhou D. (2017). Involvement of cAMP receptor protein in biofilm formation, fimbria production, capsular polysaccharide biosynthesis and lethality in mouse of *Klebsiella pneumoniae* serotype K1 causing pyogenic liver abscess. *J. Med. Microbiol.* 66 1–7. 10.1099/jmm.0.000391 27902401

[B34] Parales R. E. Harwood C. S. (1993). Construction and use of a new broad-host-range lacZ transcriptional fusion vector, pHRP309, for gram- bacteria. *Gene* 133 23–30. 10.1016/0378-1119(93)90220-w 8224891

[B35] Quincozes-Santos A. Bobermin L. D. de Assis A. M. Goncalves C. A. Souza D. O. (2017). Fluctuations in glucose levels induce glial toxicity with glutamatergic, oxidative and inflammatory implications. *Biochim. Biophys. Acta Mol. Basis Dis.* 1863 1–14. 10.1016/j.bbadis.2016.09.013 27663722

[B36] Ridley H. Lakey J. H. (2015). Antibacterial toxin colicin N and phage protein G3p compete with TolB for a binding site on TolA. *Microbiol. Sgm* 161 503–515. 10.1099/mic.0.000024 25536997 PMC4339652

[B37] Rodriguez L. Voorhies M. Gilmore S. Beyhan S. Myint A. Sil A. (2019). Opposing signaling pathways regulate morphology in response to temperature in the fungal pathogen Histoplasma capsulatum. *PLoS Biol.* 17:e3000168. 10.1371/journal.pbio.3000168 31568523 PMC6786654

[B38] Sato T. Takano A. Hori N. Izawa T. Eda T. Sato K. (2017). Role of the inner-membrane histidine kinase RcsC and outer-membrane lipoprotein RcsF in the activation of the Rcs phosphorelay signal transduction system in *Escherichia coil*. *Microbiol. Sgm* 163 1071–1080. 10.1099/mic.0.000483 28691662

[B39] Siu L. K. Yeh K. M. Lin J. C. Fung C. P. Chang F. Y. (2012). *Klebsiella pneumoniae* liver abscess: a new invasive syndrome. *Lancet Infect. Dis.* 12 881–887. 10.1016/S1473-3099(12)70205-0 23099082

[B40] Smith P. K. Krohn R. I. Hermanson G. T. Mallia A. K. Gartner F. H. Provenzano M. D. (1985). Measurement of protein using bicinchoninic acid. *Anal. Biochem.* 150 76–85. 10.1016/0003-2697(85)90442-7 3843705

[B41] Spriestersbach A. Kubicek J. Schafer F. Block H. Maertens B. (2015). Purification of his-tagged proteins. *Methods Enzymol.* 559 1–15. 10.1016/bs.mie.2014.11.003 26096499

[B42] Supek F. Bosnjak M. Skunca N. Smuc T. (2011). REVIGO summarizes and visualizes long lists of gene ontology terms. *PLoS One* 6:e21800. 10.1371/journal.pone.0021800 21789182 PMC3138752

[B43] Sycuro L. K. Pincus Z. Gutierrez K. D. Biboy J. Stern C. A. Vollmer W. (2010). Peptidoglycan crosslinking relaxation promotes *Helicobacter* pylori’s helical shape and stomach colonization. *Cell* 141 822–833. 10.1016/j.cell.2010.03.046 20510929 PMC2920535

[B44] van Teeseling M. C. F. de Pedro M. A. Cava F. (2017). Determinants of bacterial morphology: from fundamentals to possibilities for antimicrobial targeting. *Front. Microbiol.* 8:1264. 10.3389/fmicb.2017.01264 28740487 PMC5502672

[B45] Wall E. Majdalani N. Gottesman S. (2018). The Complex Rcs Regulatory Cascade. *Annu. Rev. Microbiol.* 72 111–139. 10.1146/annurev-micro-090817-062640 29897834

[B46] Wisniewski J. R. Zougman A. Nagaraj N. Mann M. (2009). Universal sample preparation method for proteome analysis. *Nat. Methods* 6 359–362. 10.1038/nmeth.1322 19377485

[B47] Wu J. H. Tsai C. G. (2005). Infectivity of hepatic strain *Klebsiella pneumoniae* in diabetic mice. *Exp. Biol. Med.* 230 757–761. 10.1177/153537020523001009 16246903

[B48] Yang D. C. Blair K. M. Salama N. R. (2016). Staying in shape: the impact of cell shape on bacterial survival in diverse environments. *Microbiol. Mol. Biol. Rev.* 80 187–203. 10.1128/MMBR.00031-15 26864431 PMC4771367

[B49] Yang Y. S. Siu L. K. Yeh K. M. Fung C. P. Huang S. J. Hung H. C. (2009). Recurrent *Klebsiella pneumoniae* liver abscess: clinical and microbiological characteristics. *J. Clin. Microbiol.* 47 3336–3339. 10.1128/JCM.00918-09 19692563 PMC2756928

[B50] Zhang M. Lv X. Y. Li J. Xu Z. G. Chen L. (2008). The characterization of high-fat diet and multiple low-dose streptozotocin induced type 2 diabetes rat model. *Exp. Diabetes Res.* 2008:704045. 10.1155/2008/704045 19132099 PMC2613511

[B51] Zhang Y. Zhao C. Wang Q. Wang X. Chen H. Li H. (2016). High prevalence of hypervirulent *Klebsiella pneumoniae* infection in China: geographic distribution, clinical characteristics, and antimicrobial resistance. *Antimicrob. Agents Chemother.* 60 6115–6120. 10.1128/AAC.01127-16 27480857 PMC5038323

[B52] Zhu J. Y. Yang Y. Han H. Betzi S. Olesen S. H. Marsilio F. (2012). Functional consequence of covalent reaction of phosphoenolpyruvate with UDP-N-acetylglucosamine 1-carboxyvinyltransferase (MurA). *J. Biol. Chem.* 287 12657–12667. 10.1074/jbc.M112.342725 22378791 PMC3339971

[B53] Zhuang Z. Song F. Martin B. M. Dunaway-Mariano D. (2002). The YbgC protein encoded by the ybgC gene of the tol-pal gene cluster of *Haemophilus influenzae* catalyzes acyl-coenzyme A thioester hydrolysis. *FEBS Lett.* 516 161–163. 10.1016/s0014-5793(02)02533-4 11959124

