# Supplementary material for: Cyclic AMP-CRP Modulates the Cell Morphology of Klebsiella pneumoniae in High-Glucose Environment
Source: Front Microbiol. 2020 Jan 21;10:2984. doi: 10.3389/fmicb.2019.02984 (PMC6985210; doi:10.3389/fmicb.2019.02984)
Supplement: Supplementary file 1 [file Table_1.DOCX]

Table S1 Oligonucleotides used in this study

| Genes name | Primers name ^a^ (Restriction enzymes) | Sequences |
| --- | --- | --- |
| Overexpression | *tolB-EcoRI*-F | TCCTGAATTCGCGATCTGAGTTCTGGTAAG |
|  | *tolB-SalI*-R | TCCTGTCGACGATATCGTACTTGTCGAGA |
|  | *rcsf-NcoI*-F | TAGACCATGGAAGCTGACGTTCATACCCG |
|  | *rcsf-pstI*-R | TATACTGCAGGAACCTGATTGTGGCGCGTG |
|  | *ftsI-NcoI*-F | TAATCCATGGGCAAACGCGTGGGTGTAAT |
|  | *ftsI-EcoRI*-R | TAATGAATTCCTCCCGTCGCGTCTTGTTG |
| qPCR | *tolB-*qF | TTGCGTATCAACTGGTGGACAC |
|  | *tolB-*qR | CCACGTAGGCGATACGGGTAC |
|  | *ybgC-*qF | TTATGAAAGAGCACGCACAGAG |
|  | *ybgC-*qR | AGAGGTGCCGCGCATCGAGGT |
|  | *Rcsf-*qF | TACCGCTGGTCACTTCGCAGC |
|  | *Rcsf-*qR | ACCGTTCCGCGATCTGGGTG |
|  | *ftsI*-qF | TTCTCCAGCCGCAGGACATCG |
|  | *ftsI*-qR | TTCTCCAGCCGCAGGACATCG |
|  | *ompA*-qF | tgttgccagtaaccgggttgg |
|  | *ompA*-qR | cgaaagacggttccgctgttg |
|  | *16S RNA*-qF | ATGACCAGCCACACTGGAAC |
|  | *16S RNA*-qR | CTTCCTCCCCGCTGAAAGTA |
| EMSA | *tolB-*EMSA-F | TGCGTATCAAACTTGCTCCTGAC |
|  | *tolB-*EMSA-R | CAGGTCAGCTGCCACGATACCA |
|  | *ftsI-* EMSA-F | TGCGAGCACTAGGCAAGTTGATG |
|  | *ftsI*- EMSA-R | AGTTGGCCTGTTCTTCCTGAC |
|  | *rcsF*- EMSA-F | tctgattaaactgaaagacgg |
|  | *rcsF*- EMSA-R | AGTATTTCCTCAATGAATAA |
| Lac*Z* | *tolB-*SalI-F | TGCCGTCGACTGCGTATCAAACTTGCTCCT |
|  | *tolB-*EcoRI -R | TAATGAATTCCAGGTCAGCTGCCACGATAC |
|  | *ftsI*- SalI-F | TGCCGTCGACTGCGAGCACTAGGCAAGTTG |
|  | *ftsI*- EcoRI-R | TAATGAATTCAGTTGGCCTGTTCTTCCTG |
|  | *rcsF*- SalI -F | TCGTGTCGACtctgattaaactgaaagacgg |
|  | *rcsF*- EcoRI -R | TAATGAATTCAGTATTTCCTCAATGAATAA |

1. Including genes name and restriction enzymes site. F, forward; R, reverse.
